# Immunomodulatory Effects of *N*-Acetyl Chitooligosaccharides on RAW264.7 Macrophages

**DOI:** 10.3390/md18080421

**Published:** 2020-08-12

**Authors:** Jun-Jin Deng, Zong-Qiu Li, Ze-Quan Mo, Shun Xu, He-Hua Mao, Dan Shi, Zhi-Wei Li, Xue-Ming Dan, Xiao-Chun Luo

**Affiliations:** 1 School of Biology and Biological Engineering, South China University of Technology, Guangzhou Higher Education Mega Center, Panyu District, Guangzhou 510006, China; 201710106376@mail.scut.edu.cn (J.-J.D.); 201721044186@mail.scut.edu.cn (Z.-Q.L.); xushun@scut.edu.cn (S.X.); 201820136288@mail.scut.edu.cn (H.-H.M.); 201720143682@mail.scut.edu.cn (D.S.); 201620133820@mail.scut.edu.cn (Z.-W.L.); 2 Institute of Animal Sciences, Guangdong Academy of Agricultural Sciences, No. 1 Dafeng Street, Wushan Road, Tianhe District, Guangzhou 510640, China; 3 Joint Laboratory of Guangdong Province and Hong Kong Regions on Marine Bioresource Conservation and Exploitation, College of Marine Sciences, South China Agricultural University, Guangzhou 510642, China; mzq1990@scau.edu.cn (Z.-Q.M.); dxm72@scau.edu.cn (X.-M.D.)

**Keywords:** *N*-acetyl chitooligosaccharides, macrophage, immune enhancement, anti-inflammation

## Abstract

The ongoing development of new production methods may lead to the commercialization of *N*-acetyl chitooligosaccharides (NACOS), such as chitosan oligosaccharides (COS). The bioactivity of NACOS, although not well detailed, differs from that of COS, as they have more acetyl groups than COS. We used two enzymatically produced NACOS with different molecular compositions and six NACOS (NACOS1–6) with a single degree of polymerization to verify their immunomodulatory effects on the RAW264.7 macrophage cell line. We aimed to identify any differences between COS and various NACOS with a single degree of polymerization. The results showed that NACOS had similar immune enhancement effects on RAW264.7 cells as COS, including the generation of reactive oxygen species (ROS), phagocytotic activity, and the production of pro-inflammation cytokines (IL-1β, IL-6, and TNF-α). However, unlike COS and lipopolysaccharide (LPS), NACOS1 and NACOS6 significantly inhibited nitric oxide (NO) production. Besides their immune enhancement effects, NACOS also significantly inhibited the LPS-induced RAW264.7 inflammatory response with some differences between various polymerization degrees. We confirmed that the NF-κB pathway is associated with the immunomodulatory effects of NACOS on RAW264.7 cells. This study could inform the application of NACOS with varying different degrees of polymerization in human health.

## 1. Introduction

Chitin is a polymer of *N*-acetylglucosamine, which is the second most abundant polysaccharide in nature after cellulose, and it is commonly found in crustacean shells, insect cuticles, and fungal cell walls [1,2]. Chitin in shrimp shells is usually tightly entangled with proteins and minerals. The production of chitin from shrimp shells traditionally entails the use of HCl for demineralization and NaOH for deproteinization [3]. The low solubility of chitin limits its applications [4]. Given the lack of commercial chitinase, chitin is deacetylated by a high concentration of NaOH (10 M) and transformed to chitosan. Chitosan is further hydrolyzed to chitosan oligosaccharides (COS) by chitosanases or non-specific glycosidases [5,6]. The COS are currently commercialized and widely used in cosmetics, animal husbandry, medicine, and food industries. Many bioactivities of COS have been reported, including immunoregulatory [7], antioxidant [8], anti-tumor [9], anti-bacterial [10], antihypertensive [11], and anti-diabetes [12] activities, among others.

The COS can act as immunostimulants to elicit immune cell responses, including the enhancement of reactive oxygen species (ROS), nitric oxide (NO) production, phagocytosis of macrophages, and the generation of pro-inflammatory cytokines, such as IL-1β, IL-6, and TNF-α, through the NF-κB, AP-1, MAPK, and PI3K/Akt signaling pathways [13,14,15]. Besides their immune enhancement effects, COS can function as anti-inflammatory agents. Pretreatment with COS could attenuate the lipopolysaccharide (LPS)-induced macrophage inflammatory response by two possible mechanisms. One mechanism facilitates the competitive binding of COS with LPS and thereby inhibits the binding of LPS to its pathogen-associated molecular pattern (PAMP) receptor (Toll-like receptor 4 (TLR4)), to diminish the corresponding inflammation signal transduction, including the NF-κB, MAPK, and other pathways [16,17]. Another mechanism is affected by reducing NF-κB nucleus translocation via interference with the OGT (O-GlcNAc transferase)-dependent O-GlcNAcylation of NF-κB [18].

Both COS and NACOS (*N*-acetyl chitooligosaccharides) are polymers connected by β-1, 4 glycoside bonds with their monosaccharides glucosamine and GlcNAc, respectively (Supplementary Figure S1). Soluble NACOSs have long demonstrated many great biological effects including antitumor [19], immunostimulatory [20], anti-inflammatory activities [21], and anti β-amyloid in Alzheimer’s disease [22], which make NACOSs great candidates of dietary supplements for human health. Compared with COS, the mechanisms underlying the bio-function of NACOS are not well documented, owing to their limited availability. The NACOS are usually prepared through the *N*-acetylation of COS by enzymatic or chemical methods; however, these methods are not very efficient and are labor intensive [23,24]. The NACOS show some similar immune promotion effects to COS. The peritoneal injection of NACOS promotes the infiltration of polymorphonuclear leucocytes and increases cell-active oxygen-generation activity [25]. *N*-acetyl chitohexaose (NACOS6) inhibits *Candida albicans* infection in the kidneys of mice [20]. The mechanisms by which NACOS enhances immune defense in mammals are also not the same as those for COS.

Some bioactivities of NACOS differ from those of COS and reportedly depend on their acetyl groups [26,27,28]. For instance, plant cells sense NACOS as a signal of fungal infection through receptors containing the lysine motif (LysM) and interact with the acetyl group of NACOS, which is absent in COS [29,30]. In animal immunomodulation, monosaccharides of chitosan and chitin, glucosamine (GlcN), and *N*-acetylated glucosamine (GlcNAc) suppress the expression of different sets of pro-inflammatory cytokines in rheumatoid arthritis (RA) mouse models [31]. Few effects of NACOS on the macrophage immune response have been reported, and their immune enhancement and anti-inflammation mechanisms have not been characterized as well as those of COS. Whether NACOS with different degrees of polymerization (DP) have different immunomodulatory effects is still not known.

Recently, an efficient recombinant chitinase, Chit46, was developed [32]. Combined with aspartic proteases, the convenient commercial enzymatic production of NACOS from shrimp shells has also been developed, omitting all chemical processing steps of traditional NACOS production. [33]. With this novel method, two NACOS samples, low DP NACOS (LNACOS) and high DP NACOS (HNACOS), are produced, and they show different immune enhancement and anti-inflammation effects. To determine whether the different immunomodulatory effects were caused by different DPs, single DP NACOS (NACOS1–6), LNACOS, HNACOS, and COS were compared for their non-specific immune stimulation and anti-LPS-induced inflammatory effects on RAW246.7 macrophages. Furthermore, NO/ROS production, phagocytosis, and pro-inflammation cytokines were detected. The underlying mechanisms behind various phenomena, including *NOX2* and *iNOS* gene expression and NF-κB pathway activation, were also verified.

## 2. Results

### 2.1. Effects of NACOS on the Non-Specific Immune Response of RAW264.7 Cells

**Figure 1:**
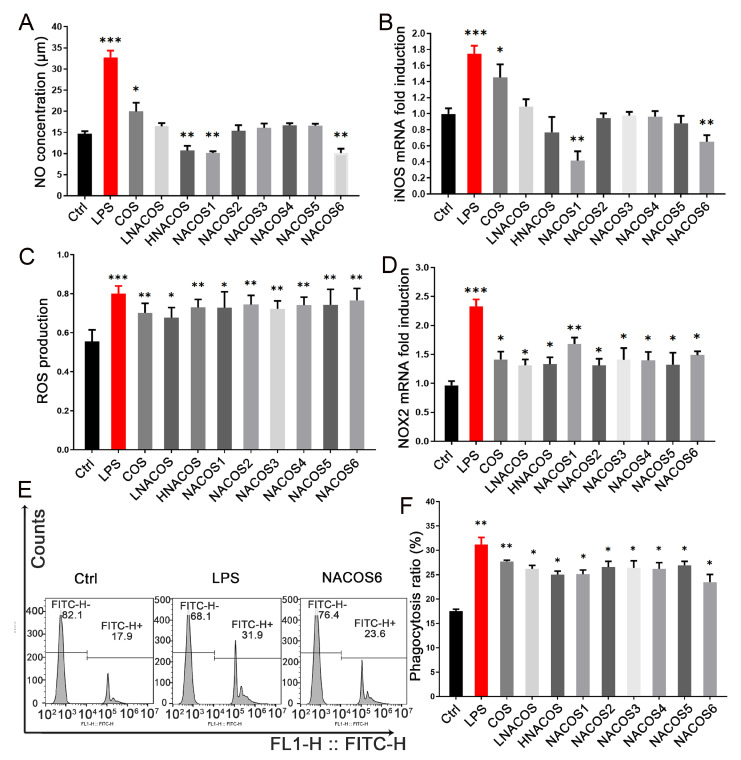
Effects of *N*-acetyl chitooligosaccharides (NACOS) on production of nitric oxide (NO), reactive oxygen species (ROS), gene expression of *iNOS* and *NOX2*, and phagocytosis of RAW264.7 cells. Cells were treated with NACOS (100 µg/mL) or lipopolysaccharide (LPS) (1 µg/mL). (**A**) NO production following treatment with NACOS for 24 h. (**B**) Expression of the *iNOS* gene following treatment with NACOS for 8 h. (**C**) ROS production following treatment with NACOS for 4 h. (**D**) Expression of the *NOX2* gene following treatment with NACOS for 4 h. (**E**) Phagocytosis of beads by RAW 264.7 cells, as detected by flow cytometry, following treatment with NACOS for 24 h. Only the control (Ctrl), LPS, and NACOS6 treatments are shown (chitosan oligosaccharides (COS) and NACOS1–5 are presented in Supplementary Figure S3). (**F**) Histogram of phagocytosis rates following treatment with NACOS. Data were normalized to that of β-actin and presented as the fold induction compared with the control group. Values are presented as the mean ± SD (*n* = 3). (* *p* < 0.05; ** *p* < 0.01; *** *p* < 0.001, compared with the control group).

The 3-(4,5-dimethyltiazol-2 yl)-2,5-diphenyl-tetrazolium bromide (MTT) assay showed that all eight NACOS samples (LNACOS, HNACOS, and single DP NACOS1–6) had no obvious cytotoxic effects on RAW264.7 macrophages (Supplementary Figure S2). To determine whether these NACOS were able to enhance the non-specific immune response of macrophages (as COS can), the NO, ROS, and phagocytosis of RAW264.7 cells were evaluated, after being treated with each of the eight NACOS samples and compared with cells treated with COS (mixtures of COS1–7) and an LPS positive control. After incubation with LPS for 24 h, the NO concentration of the RAW264.7 cell culture supernatant was doubly higher than that of the control (Figure 1A). As expected, COS also significantly increased NO concentration, which is consistent with previous reports [14].

Unlike COS, none of the NACOS samples, including LNACOS, HNACOS, and NACOS1–6 were able to increase NO production. In contrast, HNACOS, NACOS1, and NACOS6 significantly reduced NO production. Regarding the immune response, NO was produced from l-arginine and catalyzed by inducible nitric oxide synthase (iNOS) [34]. The mRNA level of *iNOS* was detected following NACOS treatment for 8 h. The iNOS expression pattern correlated with the NO concentration, as shown in Figure 1B. The iNOS expression in the HNACOS, NACOS1, and NACOS6 groups was lower than that in the control group, as evidenced by the NO concentration; however, only the NACOS1 and NACOS6 groups showed significant differences.

After macrophages phagocytose pathogens, ROS are important bactericides [35]. The ROS inside RAW264.7 cells were evaluated, and the results showed that all NACOS samples were able to improve ROS production in a similar manner to COS, after treatment for 4 h, but they were not as effective as LPS (Figure 1C). The key enzyme for ROS production is NADPH oxidase 2 (NOX2) [36], and its expression was significantly upregulated in all macrophages treated with NACOS for 4 h (Figure 1D), which is consistent with the trend in ROS production.

The phagocytosis rate of fluorescent beads among the RAW264.7 cells was detected by flow cytometry after treatment with NACOS for 24 h, and it was found to be significantly increased in all groups, as well as in the COS and LPS groups (Figure 1E,F, and Supplementary Figure S3). No significant differences were noted in ROS production or phagocytosis among the HNACOS, LNACOS, NACOS1–6, or COS groups. These data proved that NACOS could improve macrophage ROS production and phagocytosis in the innate immunity response.

### 2.2. NACOS Promoted the Production of Pro-Inflammatory Cytokines via the NF-κB Pathway

**Figure 2:**
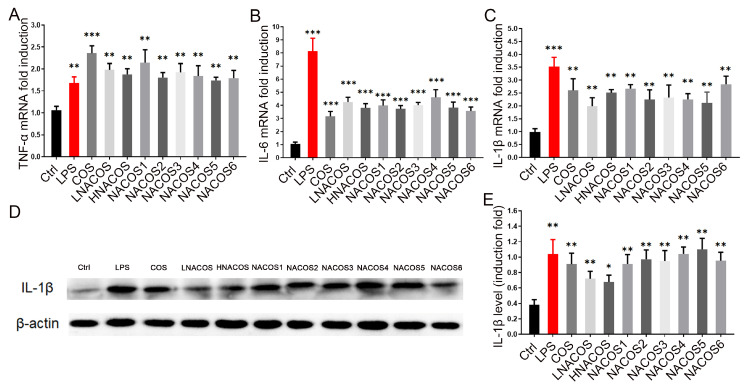
Effects of *N*-acetyl chitooligosaccharides (NACOS) on gene expression of *TNF-α*, *IL-6*, *IL-1β*, and protein level of IL-1β in RAW264.7 macrophages. The RAW264.7 cells were treated with NACOS (100 µg/mL) or LPS (1 µg/mL) for 8 h. Gene expressions were measured by RT-qPCR. Gene expressions of (**A**) *TNF-α*; (**B**) *IL-6*; and (**C**) *IL-1β*, following treatment with NACOS. (**D**) Western blot analysis of IL-1β following treatment with NACOS for 8 h. (**E**) IL-1β protein levels following treatment with NACOS for 8 h, evaluated by densitometry analysis of the blot. Data were normalized to that of β-actin and are presented as the fold induction compared with the control group. Values are presented as the mean ± SD (*n* = 3). * *p* < 0.05; ** *p* < 0.01; *** *p* < 0.001, compared with the control group.

In the macrophage inflammatory response, NO production is usually promoted accompanied by ROS production and an increase in the phagocytosis rate [37]. As described above, NACOS reduced NO production, but it increased ROS production and the phagocytosis rate. To confirm whether NACOS induced the macrophage inflammatory response, the mRNA levels of three pro-inflammatory cytokines, TNF-α, IL-6, and IL-1β, were evaluated through RT-qPCR after 8 h of treatment. The results showed that the expression of all three pro-inflammatory genes were significantly up-regulated in all NACOS groups, as they were in the COS and LPS groups (Figure 2A–C). No significant differences were noted among the eight NACOS groups. To confirm whether the protein levels of pro-inflammation cytokines were also promoted in line with their mRNA levels, Western blot analysis of IL-1β in the cell lysates was conducted. The results showed that the IL-1β protein levels were increased, as was the mRNA expression (Figure 2D,E).

**Figure 3:**
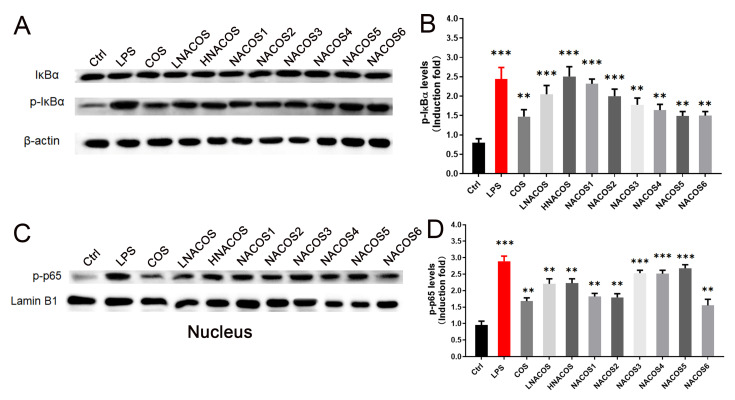
Effects of *N*-acetyl chitooligosaccharides (NACOS) on the phosphorylation of IκBα and NF-κB p65 in RAW264.7 macrophages. The RAW 264.7 cells were treated with similar concentrations of the various NACOS (100 µg/mL), chitosan oligosaccharides (COS) (100 µg/mL), or lipopolysaccharide (LPS) (1 µg/mL) for 8 h. (**A**) Western blot analysis of cellular IκBα and phosphorylated IκBα. (**B**) Cellular phosphorylated IκBα protein levels based on densitometry analysis of the blot. (**C**) Western blot analysis of nucleic phosphorylated NF-κB p65. (**D**) Nucleic phosphorylated NF-κB p65 protein levels based on densitometry analysis of the blot. Data were normalized to β-actin or Lamin B1 and presented as the induction fold, compared with the control group. Values are presented as the mean ± SD (*n* = 3). ** *p* < 0.01; *** *p* < 0.001, compared with the control group.

The COS reportedly promote the macrophage immune response through the NF-κB pathway [15]. To determine whether NACOS also affect macrophages by this pathway, Western blot analysis of two-terminal proteins in this pathway, IκBα and p65, was conducted. The total protein levels of IκBα in the RAW264.7 cytoplasm were similar among all NACOS groups, as well as the LPS, COS, and phosphate-buffered saline (PBS) control groups (Figure 3A). Furthermore, the phosphorylated IκBα levels were significantly up-regulated in all groups, compared with the PBS control, which indicated that all NACOS types caused the phosphorylation of IκBα, just as the LPS and COS groups did.

Phosphorylated IκBα became detached from the heterodimer of the transcription factor, NF-κB (p65/p50). The exposed p65 was further phosphorylated, entered the nucleus, and became bound to the promoter region of the downstream pro-inflammatory genes. Western blot analysis showed that the levels of p-p65 in the nucleus were increased among all eight NACOS groups, as well as the LPS and COS groups. These data confirmed that NACOS could promote the production of pro-inflammatory cytokines by RAW264.7 through the NF-κB pathway.

### 2.3. NACOS Inhibited the LPS-Induced RAW264.7 Inflammatory Response

**Figure 4:**
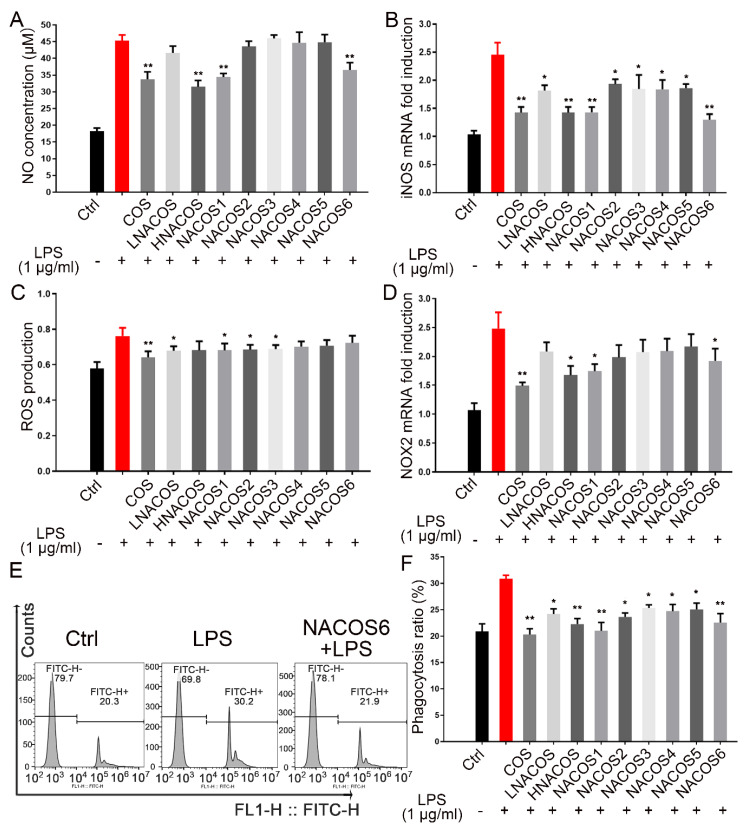
Effects of *N*-acetyl chitooligosaccharides (NACOS) pretreatment on the production of NO, reactive oxygen species (ROS), gene expression of *iNOS* and *NOX2*, and phagocytosis in lipopolysaccharide (LPS)-treated RAW264.7 macrophages. Cells were pretreated with NACOS (100 µg/mL), chitosan oligosaccharides (COS) (100 µg/mL), or lipopolysaccharide (LPS) (1 µg/mL) for 24 h and then co-incubated with LPS. Gene expressions were measured by RT-qPCR. (**A**) Production of NO following treatment with NACOS for 24 h and LPS for 24 h. (**B**) Expression of the *iNOS* gene following treatment with NACOS for 24 h and LPS for 8 h. (**C**) ROS production following treatment with NACOS for 24 h and LPS for 4 h. (**D**) Expression of the *NOX2* gene following treatment with NACOS for 24 h and LPS for 4 h. (**E**) Phagocytosis by RAW 264.7 cells, as detected by flow cytometry following treatment with NACOS for 24 h and LPS for 4 h. Phagocytosis of beads was determined by flow cytometry. Only the control (Ctrl), LPS, and NACOS6 + LPS treatments are shown (COS + LPS and NACOS1 to NACOS5 + LPS are presented in Supplementary Figure S4). (**F**) Histogram of phagocytosis rates following pretreatment of RAW264.7 cells with NACOS. Data were normalized to β-actin and presented as the fold induction, compared with the control group. Values are presented as the mean ± SD (*n* = 3). * *p* < 0.05; ** *p* < 0.01, compared with the only LPS-treated group.

Pre-incubation of RAW264.7 with COS could inhibit the LPS-induced inflammatory response [38,39]. Therefore, to determine whether NACOS have a similar effect to COS, RAW264.7 cells were pre-incubated with COS, LNACOS, HNACOS, and NACOS1–6 for 24 h, before they were treated with LPS. The NO and ROS production, *iNOS* and *NOX2* gene expression, and phagocytosis by RAW246.7 were assessed, as described above. The results showed that pretreatment with COS, HNACOS, NACOS1, and NACOS6 was able to significantly reduce LPS-induced NO production (Figure 4A). The *iNOS* expression in the COS and all eight NACOS pretreatment groups was significantly lower than that in the direct LPS treatment group (Figure 4B).

Regarding ROS production, COS, LNACOS, NACOS1, NACOS2, and NACOS3 pretreatments were found to significantly reduce LPS-induced ROS production (Figure 4C). Expression of the *NOX2* gene in COS and all NACOS pretreated groups seemed to have been lower than that in the direct LPS treatment group; however, only the COS, HNACOS, and NACOS1 groups showed significant differences (Figure 4D). All COS and NACOS pretreatments significantly reduced the LPS-induced RAW264.7 fluorescent bead phagocytosis rate, according to counts in flow cytometry (Figure 4E,F).

### 2.4. NACOS Inhibited LPS-Induced Inflammation through the NF-κB Pathway

**Figure 5:**
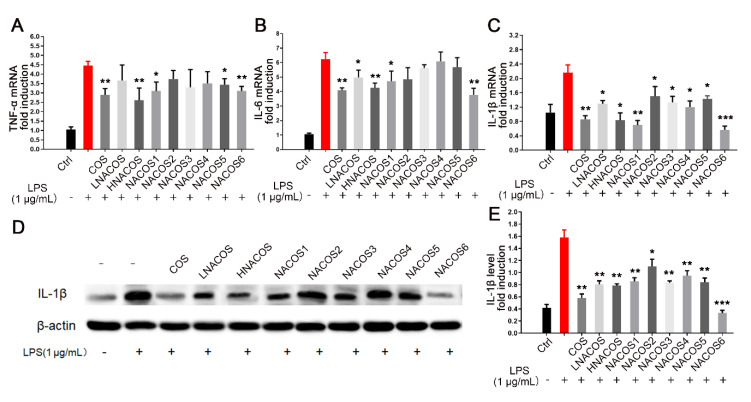
Effects of *N*-acetyl chitooligosaccharides (NACOS) pretreatment on gene expression of *TNF-α*, *IL-6*, *IL-1β*, and *IL-1β* and protein levels in lipopolysaccharide (LPS)-induced RAW264.7 macrophages. RAW-264.7 cells were pretreated with NACOS (100 µg/mL), chitosan oligosaccharides (COS) (100 µg/mL), or LPS (1 µg/mL) for 24 h, and then further co-incubated with LPS (1 µg/mL). Gene expressions were measured by RT-qPCR. Gene expressions of (**A**) *TNF-α*; (**B**) *IL-6*; and (**C**) *IL-1β* following treatment with NACOS for 24 h and LPS for 8 h. (**D**) Western blot analysis of IL-1β following treatment with NACOS for 24 h and LPS for 8 h. (**E**) The IL-1β levels based on densitometry analysis of the blot. Values are presented as the mean ± SD (*n* = 3). * *p* < 0.05; ** *p* < 0.01; *** *p* < 0.001, compared with the only LPS-treated group.

In addition to inhibition of the LPS-induced innate immunity response, as described above, expression of the pro-inflammatory cytokine genes was detected. The mRNA levels of *TNF-α*, *IL-6*, and *IL-1β* in the COS group and all NACOS pretreatment groups were much lower than those in the LPS direct treatment group; however, mRNA levels of *TNF-α* in the NACOS2, NACOS3, and NACOS4 pretreatment groups, and those of *IL-6* in the NACOS2, NACOS3, NACOS4, and NACOS5 pretreatment groups showed no significant differences (Figure 5A–C). Western blot analysis of cellular IL-1β protein in all pretreatment groups showed significantly lower levels, compared with the LPS direct treatment group (Figure 5D,E).

**Figure 6:**
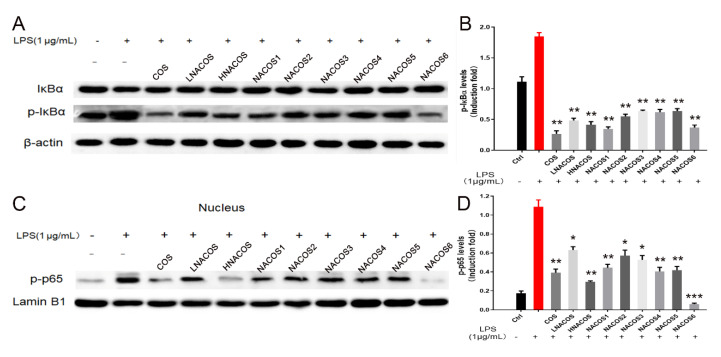
Effects of *N*-acetyl chitooligosaccharides (NACOS) pretreatment on the phosphorylation of IκBα and NF-κB p65 in lipopolysaccharide (LPS)-induced RAW264.7 macrophages. RAW 264.7 cells were pretreated with the similar concentrations of NACOS (100 µg/mL), chitosan oligosaccharides (COS) (100 µg/mL), or LPS (1 µg/mL) for 24 h and then co-incubated with LPS for 8 h. (**A**) Western blot analysis of cellular IκBα and phosphorylated IκBα. (**B**) Cellular phosphorylated IκBα protein levels based on densitometry analysis of the blot. (**C**) Western blot analysis of nucleic phosphorylated NF-κB p65. (**D**) Nucleic phosphorylated NF-κB p65 protein levels based on densitometry analysis of the blot. Data were normalized to β-actin or Lamin B1, and presented as the induction fold, compared with the control group. Values are presented as the mean ± SD (*n* = 3). * *p* < 0.05; ** *p* < 0.01; *** *p* < 0.001, compared with the only LPS-treated group.

The mRNA and protein levels in the NACOS6 pretreatment group were notably even lower than those in the PBS pretreatment group. Levels of cellular p-IκBα in the COS and all NACOS pretreatment groups were significantly lower than they were in the LPS direct treatment group and the PBS pretreatment group (Figure 6A,B). Levels of nucleic p-p65 in the COS and all NACOS pretreatment groups were significantly lower than those in the LPS direct treatment group, and those in the NACOS6 pretreatment group were lower than those in the PBS pretreatment control (Figure 6C,D). These data all show that NACOS could inhibit LPS-induced inflammation by regulating the NF-κB pathway. Among eight NACOS samples, HNACOS, NACOS1, and NACOS6 showed stronger inhibitory effects than the others, regardless of the levels of NO production, pro-inflammatory cytokine production, or phosphorylation of IκBα and p65. These findings indicate that NACOS with different DP show varying effects on immune modulation.

## 3. Discussion

The NACOS generally have not been as commercialized as COS, owing to the high cost associated with the process of re-acetylation from COS by chemical or enzymatic methods [40]. As more efficient chitinases continue to be developed, the traditional methods of NACOS production through chitin deacetylation, the hydrolysis of chitosan, and the re-acetylation of COS would be abandoned and replaced by the direct chitinase hydrolysis of chitin or crustacean shells, which will would facilitate the imminent commercialization of NACOS [32,33]. Until now, the bioactivities of NACOS and their underlying mechanisms have been poorly understood and lagged far behind our understanding of COS mechanisms. The bioactivities of NACOS differ between samples with different mixtures of the DP, and the exact functions of single DP NACOS are also not well verified [28,38]. We used the RAW264.7 macrophage cell line, an important immune cell line, to verify its innate immunity and inflammatory response against two NACOS mixtures and six single DP NACOS (1–6) samples and compared their effects with those of a COS mixture and LPS.

For non-specific immune responses, the improvements observed in macrophage ROS production, *NOX2* gene expression, and phagocytosis following treatment with NACOS were similar to those following treatment with COS. The COS can act as PAMP molecules and promote the expression of macrophage pro-inflammatory cytokines through activation of the NF-κB, MAPK, PI3K/Akt-3, and AP-1 pathways [14,15]. The effects of the corresponding pattern recognition receptors (PRRs) have not been confirmed, and chitin PRRs are believed to be Dectin-1, TLR-2, FIBCD, the mannose receptor, and RegIIIc [41]. The NACOS and COS may use similar PRRs to activate macrophage innate immunity. In the present study, we confirmed that NACOS could activate NF-κB pathways by promoting the phosphorylation and nuclear entry of p65. Whether NACOS could activate the MAPK, PI3K/Akt-3, and AP-1 pathways requires further study.

During the process of macrophage activation, NO production is usually accompanied by ROS production and the secretion of pro-inflammatory cytokines [42]. In the present study, unlike LPS and COS, none of the eight NACOS samples were able to enhance NO production, and HNACOS, NACOS1, and NACOS6 significantly downregulated both *iNOS* gene expression and NO production. HNACOS contained 13.8% NACOS6 and no NACOS1. This finding suggests that the NO downregulatory effects of HNACOS were due to its NACOS6 content. Mice iNOS are regulated by various transcription factors, including NF-κB, STAT-1α, AP-1, HIF-1, and STAT3, among others [43]. We confirmed the activation NF-κB; however, the downregulation of iNOS expression is likely regulated by other transcription factors. We do not yet know which pathways were regulated by NACOS or the specific mechanisms by which they exerted effects that differed from those of COS. The reasons for the strong inhibitory effects of NACOS1 and NACOS6 on NO production, which were not evident with NACOS2–5, requires further study.

Besides their enhancement of innate immunity in macrophages, NACOS showed similar anti-LPS-induced macrophage inflammatory effects, similar to COS. The anti-inflammatory effects of COS are based on different mechanisms related to their innate immunity enhancement effects. The amino groups of COS confer a positive charge and facilitate their binding to negative LPS. In addition, the hydroxyl groups of COS provide protons for the formation of hydrogen bonds, and *N*-acetyl groups participate in hydrophobic interactions with LPS. These interactions all promote COS binding to LPS and block LPS binding to its PAMP receptor, TLR4 [44,45], which further inhibits the LPS-induced inflammatory effects. The NACOS have higher acetylation levels and a less positive charge than the COS. However, NACOS have a similar hydroxyl group composition and more *N*-acetyl groups, which make them more likely (than COS) to interact with LPS.

Another mechanism of the anti-LPS-induced inflammatory effects of COS is their ability to inhibit O-GlcNAcylation to reduce NF-κB nuclear localization. Over the last decade, O-GlcNAcylation has been found to be an important post-translation modification. The GlcNAcylation of transcription factors can regulate cellular processes and the addition of GlcN, GlcNAc, COS, and NACOS can improve these effects. The protein GlcNAcylation levels of different cells affect their cell responses through different pathways, including T/B cell activation [46,47], macrophage activation [48], chondrocyte inflammatory responses [49,50], and vascular endothelial inflammatory responses [18], among others.

Furthermore, when carbohydrates enter the hexosamine biosynthetic pathway, they are converted to Uridine diphosphate N-acetylglucosamine (UDP-GlcNAc) and catalyzed by OGT to become the glycosyl part of the protein. In addition, GlcN could reportedly inhibit LPS-induced RAW264.7 activation of the NF-κB pathway by increasing levels of protein O-GlcNAcylation [51]. GlcN competes with glucose and engages membrane glucose transporters (GLUTs) in mammalian cells, while GlcNAc is transported by unknown transporters that differ from GLUTs [50]. The mechanisms by which COS and NACOS are imported into cells with specific transporters are still not known. Based on previous reports and reductions of nuclear localization of p-p65 in the present study, NACOS may affect innate immune enhancement by RAW264.7 and the anti-inflammatory response by affecting the GlcNAcylation level in the cell.

A previous study reported that different DP compositions of COS have different effects on mammalian cells; e.g., COS with an average molecular weight of 3 kDa (about DP15) can more strongly boost nucleic p65 levels than those with a molecular weight of 50 kDa (about DP250) [15]. Few studies have compared the effects of single DP COS or NACOS. The positive charges of COS from amino groups make them more soluble than the less charged NACOS.

In the present study, we first compared the immunomodulatory effects of NACOS with DP1–6 on macrophages. We found that NACOS with different DP had different inhibitory effects on NO production; NACOS1 and NACOS6 had stronger inhibition than others, even though their ROS production and phagocytotic enhancement showed no significant differences. It is to be noted that in the anti-LPS-induced inflammation test, NACOS1 and NACOS6 also showed better anti-inflammation effects than other DP NACOSs. Between them, NACOS6 resulted in lower IL-1β and nucleic phosphorylated p65 levels than NACOS1. The transport of GlcNAc and NACOS into plant cells is facilitated by various transporters, and only NACOS with a DP higher than 6 could cause cross-linking and activate the lysine motif (LysM) and motif-containing receptors to modulate host plant immunity. The mechanisms of transport and the O-GlcNAcylation effects of various DP NACOS in mammalian cells are still not known. NACOS1 were reported to be absorbed by an unknown transporter different from glucose transporter GLUT in mammalian cells and increased cellular UDP-GlcNAc levels and protein O-GlcNAcylation [52] and N-glycan branching [53]. No transporter has been identified for NACOS2-6 in mammals, and it is not clear whether they could directly be transported into mammalian cells. The possible mammalian membrane receptors of NACOS, including Dectin-1 and mannose receptors, need further confirmation. We believe that NACOS1 is more readily absorbed than NACOS6, while NACOS6, but not NACOS1, may cross-talk with membrane receptors. NACOS1 and NACOS6 may employ different mechanisms in their immune modulation process. In future studies, isotype labeled NACOS, such as ^13^C_6_-GlcNAc, can be used to trace NACOS’s receptors, cell transportation, and cellular glycosylation. Proteomic analysis of O-GlcNAcylation of cellular or nucleic proteins could be carried out to identify the glycosylation modification of transcription factors. Whether different DP NACOS have different effects on other mammalian cells and the associated underlying mechanisms need further study.

In conclusion, we found that NACOS with different DP enhance innate immunity and exert anti-LPS-induced macrophage inflammatory effects similar to COS. These findings suggest the potential use of NACOS as immunomodulatory agents. The NACOS exert effects that differ somewhat from those of COS, owing to its higher number of acetyl groups. These differing effects indicate novel useful bioactivities of NACOS, which are not observed with COS. Besides the immunomodulatory function, NACOS has other bioactivities, such as anti-tumor and anti-Alzheimer’s disease, which make it a good candidate for use as a dietary supplement. This study may serve as a reference for the application and continued commercialization of enzymatically produced NACOS in the human health industry.

## 4. Materials and Methods

### 4.1. Materials and Reagents

The COS mixture was obtained from Changlong Biotechnology Co. Ltd. (Shenzhen, China) (Supplementary Figure S5). *N*-acetyl glucosamine (NACOS1), MTT, LPS (from *Escherichia coli* O55:B5), and dimethyl sulfoxide (DMSO) were obtained from Sigma-Aldrich Co. (St. Louis, MO, USA). The *N*-acetyl chitooligosaccharides standards (NACOS2 to NACOS6) were obtained from Megazyme (Bray, Ireland). The NACOS with a low DP (LNACOS) were prepared from colloidal chitin by the chitinase, Chit46, as previously reported [32]. The NACOS with a high DP (HNACOS) were prepared from shrimp shells [33]. The composition and de-acetylation degree (DA) of COS, LNACOS, and HNACOS are listed in Table 1.

Fetal bovine serum (FBS) and Dulbecco’s modified Eagle’s medium (DMEM) were obtained from Gibco BRL (Life Technologies, Shanghai, China). Primary antibodies to IL-1β, IκBα, phospho-specific IκBα, p65, and phospho-specific p65 were all obtained from Cell Signaling Technology (Beverly, MA, USA). The primary antibody to β-actin and Lamin B1 were obtained from Abcam (Cambridge, MA, USA). Horseradish peroxidase-labeled goat anti-rabbit and goat anti-mouse antibodies were obtained from Abcam (Cambridge, MA, USA). All other chemicals and reagents were obtained from Shanghai Sangon Biological Engineering Technology and Services Co. Ltd. (Shanghai, China). The RAW264.7 macrophages were obtained from the Shanghai Institute of Biochemistry and Cell Biology of the Chinese Academy of Science (Shanghai, China).

### 4.2. Cell Culture and Treatment

The RAW264.7 macrophages were cultured in DMEM medium supplemented with 10% FBS, 100 µmol/L penicillin, and 100 µmol/L streptomycin. Cells were maintained at 37 °C with 5% CO_2_ in a humidified atmosphere. All cells were cultured for about 48 h to reach the logarithmic phase and then used for subsequent experiments. The cells were divided into two parts—one part was treated with 100 µg/mL COS, LNACOS, HNACOS, NACOS1, NACOS2, NACOS3, NACOS4, NACOS5, or NACOS6 (the non-LPS group), while the other part was first pretreated with 100 µg/mL COS or NACOS for 24 h and then co-incubated with 1 µg/mL LPS (the LPS-treated group).

### 4.3. Cell Viability Assay

The cytotoxic effects of COS and NACOS on the viability of RAW264.7 macrophage cells were determined by the MTT assay. The control group was regarded as having 100% cell viability. Briefly, RAW264.7 macrophage cells were plated in 96-well culture plates at a density of 1 × 10^5^ cells per well and cultured overnight. Subsequently, 100 µg/mL of either COS and NACOS was introduced to the culture, and the cells were incubated for a further 24 h. The culture supernatants were discarded, and 5 mg/mL of the MTT solution was added to each well. After incubation for 4 h, the medium was carefully aspirated from each well, and 100 µL DMSO was added to dissolve the formazan crystals. The absorbance in each well was measured at 570 nm using a microplate reader (Varioskan Lux, Thermo Scientific, Waltham, MA, USA).

### 4.4. Measurement of NO Production

The NO content of the cell culture medium was measured, using the Griess reagent [1% sulfanilamide and 0.1% *N*-(1-naphthyl)–ethylenediamine dihydrochloride in 5% phosphoric acid]. The RAW264.7 cells (2 × 10^5^ cells/mL) were plated on 96-well plates (100 µL/well) and cultured for 24 h. Then, cells were incubated for 24 h in the presence of 100 µg/mL COS, NACOS, or 1 µg/mL LPS. The LPS-treated group of RAW264.7 cells were pretreated with 1 × PBS, 100 µg/mL of COS, or NACOS for 24 h in serum-free DMEM medium, followed by co-culture with 1 µg/mL LPS (positive control) for an additional 24 h. Then, a volume of 50 µL of Griess reagent was added to the culture supernatants. The absorbance was read at 540 nm, and the NO concentration was calculated using a standard curve of NaNO_2_.

### 4.5. Measurement of ROS Production

The ROS production of RAW264.7 macrophages was determined using the nitro blue tetrazolium (NBT) reduction test. The RAW264.7 cells (2 × 10^5^ cells/mL) were plated on 96-well plates (100 µL/well) and cultured for 24 h. Cells were incubated for 4 h in the presence of 100 µg/mL COS, or NACOS, or 1 µg/mL LPS. Moreover, the LPS-treated group of RAW264.7 cells were pretreated with 1 × PBS, or 100 µg/mL of COS or NACOS for 24 h, followed by co-culture with 1 µg/mL LPS in each well for 4 h. The cells were further incubated with 200 µL of 1 × PBS, containing 0.1% of NBT (Sigma, USA) and 2 µg/mL phorbol 12-myristate 13-acetate (PMA) at 37 °C for 0.5 h. The reaction was terminated with 80 µL of 70% methanol. Then, the cells were air-dried, and the blue formazan produced was dissolved in 120 µL of 2M KOH and 140 µL of DMSO. The absorbance was measured by a spectrophotometer at 620 nm, with KOH/DMSO as the blank.

### 4.6. Phagocytosis Assay

The RAW264.7 macrophage cells (4 × 10^5^ cells/ell) were seeded onto glass coverslips in a 12-well plate and allowed to adhere to the coverslips for 12 h. Then, cells were incubated for 24 h in the presence of 100 µg/mL COS, NACOS, or 1 µg/mL LPS. Moreover, the LPS-treated group of RAW264.7 cells were pretreated with 1 × PBS, or 100 µg/mL of COS or NACOS for 24 h, followed by co-culture with 1 µg/mL LPS in each well for 4 h. Then, cells were incubated with FluoSpheres™ Polystyrene Microspheres (Thermo Fisher, Waltham, MA, USA) at a multiplicity of infection of 5 at 37 °C for 1.5 h. Cells were washed three times with PBS to remove the undevoured beads. Then, the cells were analyzed with a flow cytometer (Beckon, Brea, CA, USA). The extent of phagocytosis was analyzed, using a FlowJo system. The phagocytosis rate was calculated as the percentage of counts of cells engulfing microspheres in the gate of higher florescence value (counts of right peak) to all cell counts (sums of none-engulfing cells in left peak and engulfing cells in the right peak) [54].

### 4.7. Quantitative Real-Time PCR (qPCR)

The RAW264.7 macrophage cells were treated with 100 µg/mL COS or NACOS, or 1 µg/mL LPS for 8 h, while the LPS-treated group of RAW264.7 cells was pretreated with 1 × PBS and 100 µg/mL of COS or NACOS for 24 h, followed by co-culture with 1 µg/mL LPS for 8 h. Total RNA was extracted from RAW264.7 macrophages using the TRIZOL reagent (TaKaRa, Dalian, China), according to the manufacturer’s protocol. Real-time PCR was performed on a LightCycler96 real-time system (Roche, Germany), using the SYBR Green Real-time PCR Master Mix (Thermo Fisher, USA), according to manufacturer’s instructions. Expression of the target gene was normalized to the housekeeping gene, β-actin, and calculated using the 2^−ΔΔCt^ method. The primers for qPCR are listed in Table 2.

### 4.8. Western Blotting Analysis

The RAW264.7 macrophage cells were treated with 100 µg/mL COS or NACOS, or 1 µg/mL LPS for 8 h, while the LPS-treated group of RAW264.7 cells were pretreated with 1 × PBS and 100 µg/mL of COS or NACOS for 24 h, followed by co-culture with 1 µg/mL LPS for 8 h. The cells were washed three times with cold PBS and lysed with nuclear and cytoplasmic extraction reagents (Applygen, Beijing, China). Then, the cells were subjected to centrifugation (10,000× *g*) at 4 °C for 10 min. The protein contents were measured with the BCA protein assay kit (Sangon Biotech, Shanghai, China), using bovine serum as the standard.

The samples were separated by sodium dodecyl sulfate-polyacrylamide gel electrophoresis (SDS-PAGE) and proteins were transferred onto a polyvinylidene difluoride membrane by electroblotting (Millipore, Bedford, MA, USA). After the membrane was blocked with 5% skim milk containing Tris-buffered saline (20 mM Tris-HCl, 500 mM NaCl, and 0.05% Tween-20) for 1 h at 37 °C, the blots were incubated with mouse monoclonal antibody anti-IκBα (1:500, CST); anti-p-IκBα (1:500, CST); anti-IL-1β (1:1000, CST); anti-β-actin (1:1000, Abcam); rabbit monoclonal antibody anti-NF-κB p65 (1:500,CST); anti-NF-κB p-p65 (1:500, CST); and anti-Lamin B1 (1:1000, Abcam) overnight at 4 °C. Following incubation with an appropriate secondary antibody (horseradish peroxidase-conjugated goat anti-mouse or anti-rabbit Immunoglobulin G for 1 h, proteins were detected using an ECL chemiluminescence detection kit (Sigma-Aldrich, St. Louis, MO, USA), and scanned. Protein expression was corrected depending on the amount of β-actin or Lamin B1 present in a given sample.

### 4.9. Statistical Analysis

All data were expressed as the mean ± SD of triplicate samples and were representative of at least three separate experiments. Statistical comparisons were made using the Student’s *t*-test and one-way AVOVA, followed by the Tukey’s post hoc test. *p*-values < 0.05 were considered to represent a statistically significant difference.

## Figures and Tables

**Table 1:** The composition and de-acetylation degree (DA) of COS, low degrees of polymerization NACOS (LNACOS) and high degrees of polymerization NACOS (HNACOS). DP: degrees of polymerization.

Samples	Composition (%)							DA (%)
	DP1	DP2	DP3	DP4	DP5	DP6	DP7	
COS	7.5	11.0	21.9	25.9	18.1	8.7	3.1	>95
LNACOS	1.5	94.8	2.8	0.9	-	-	-	8.7
HNACOS	-	37.4	10.4	32.3	6.1	13.8	-	8.6

**Table 2:** Primers used in this study.

Genes	Forward Primer (5′ 3′)	Reverse Primer (5′ 3′)
*iNOS*	GGTAGTAGTAGAATGGAGATAGG	CTACCTAAGATAGCAGTTGATG
*NOX2*	ACCAGACAGACTTGAGAATG	GCTGTGCTATGTTGCTCTAG
*IL-1β*	ATCTCGCAGCAGCACATC	CCAGCAGGTTATCATCATCATC
*TNF-α*	CACGCTCTTCTGTCTACTG	ACTTGGTGGTTTGCTAC
*IL-6*	AATTAAGCCTCCGACTTGTG	CACGCTCTTCTGTCTACTG
*β-actin*	GCTGTGCTATGTTGCTCTAG	TCGTTGCCAATAGTGATGAC

## Supplementary Materials

The following are available online at https://www.mdpi.com/1660-3397/18/8/421/s1, Supplementary Figure S1: Structure of NACOS and COS, Supplementary Figure S2: Effects of *N*-acetyl chitooligosaccharides (NACOS) on cell viability of RAW264.7 macrophages. RAW264.7 macrophages were treated with NACOS (100 µg/mL) for 24 h, Supplementary Figure S3: Phagocytosis rates following the treatment of NACOS. a, b, c, d, e, f, g, h, i, j, and k represent the control (Ctrl), LPS, chitosan oligosaccharide (COS), low DP NACOS (LNACOS), high DP NACOS (HNACOS), NACOS1, NACOS2, NACOS3, NACOS4, NACOS5, and NACOS6, respectively, Supplementary Figure S4: The RAW264.7 phagocytosis rates in the *N*-acetyl chitooligosaccharides (NACOS) pretreated by LPS stimulation. a, b, c, d, e, f, g, h, i, j, and k represents the control (Ctrl), LPS chitosan oligosaccharide (COS), low DP NACOS (LNACOS), high DP NACOS (HNACOS), NACOS1, NACOS2, NACOS3, NACOS4, NACOS5, and NACOS6 pretreatment groups, respectively, Supplementary Figure S5: High-performance liquid chromatography diagram of the chitosan oligosaccharide sample provided by Changlong Company.
