# Supplementary material for: Immunomodulatory Effects of N-Acetyl Chitooligosaccharides on RAW264.7 Macrophages
Source: Mar Drugs. 2020 Aug 12;18(8):421. doi: 10.3390/md18080421 (PMC7460392; doi:10.3390/md18080421)
Supplement: Supplementary file 1 [file marinedrugs-18-00421-s001.pdf]

Immunomodulatory Effects of *N*-acetyl Chitooligosaccharides on RAW264.7 Macrophages

Supplementary materials:

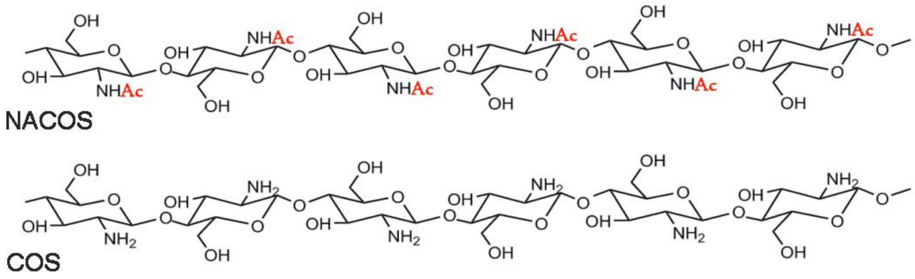

Supplementary Figure 1. Structure of NACOS and COS.

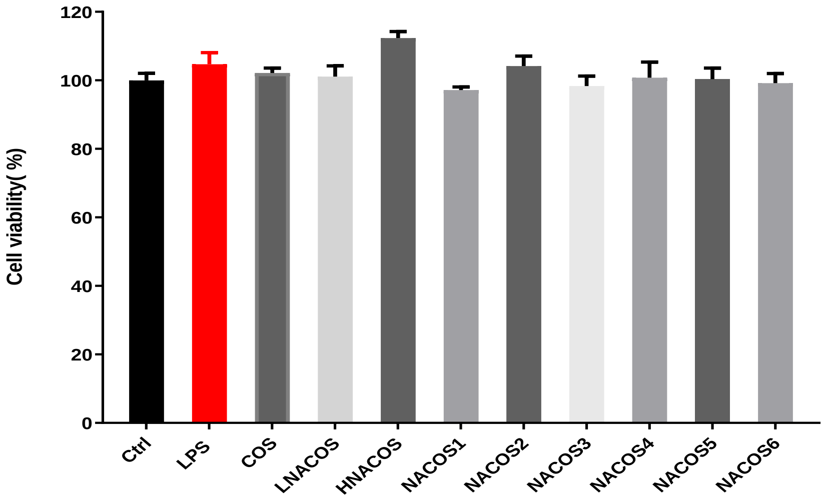

Supplementary Figure 2. Effects of *N*-acetyl chitooligosaccharides (NACOS) on cell viability of RAW264.7 macrophages. RAW264.7 macrophages were treated with NACOS (100 μg/mL）for 24 h.

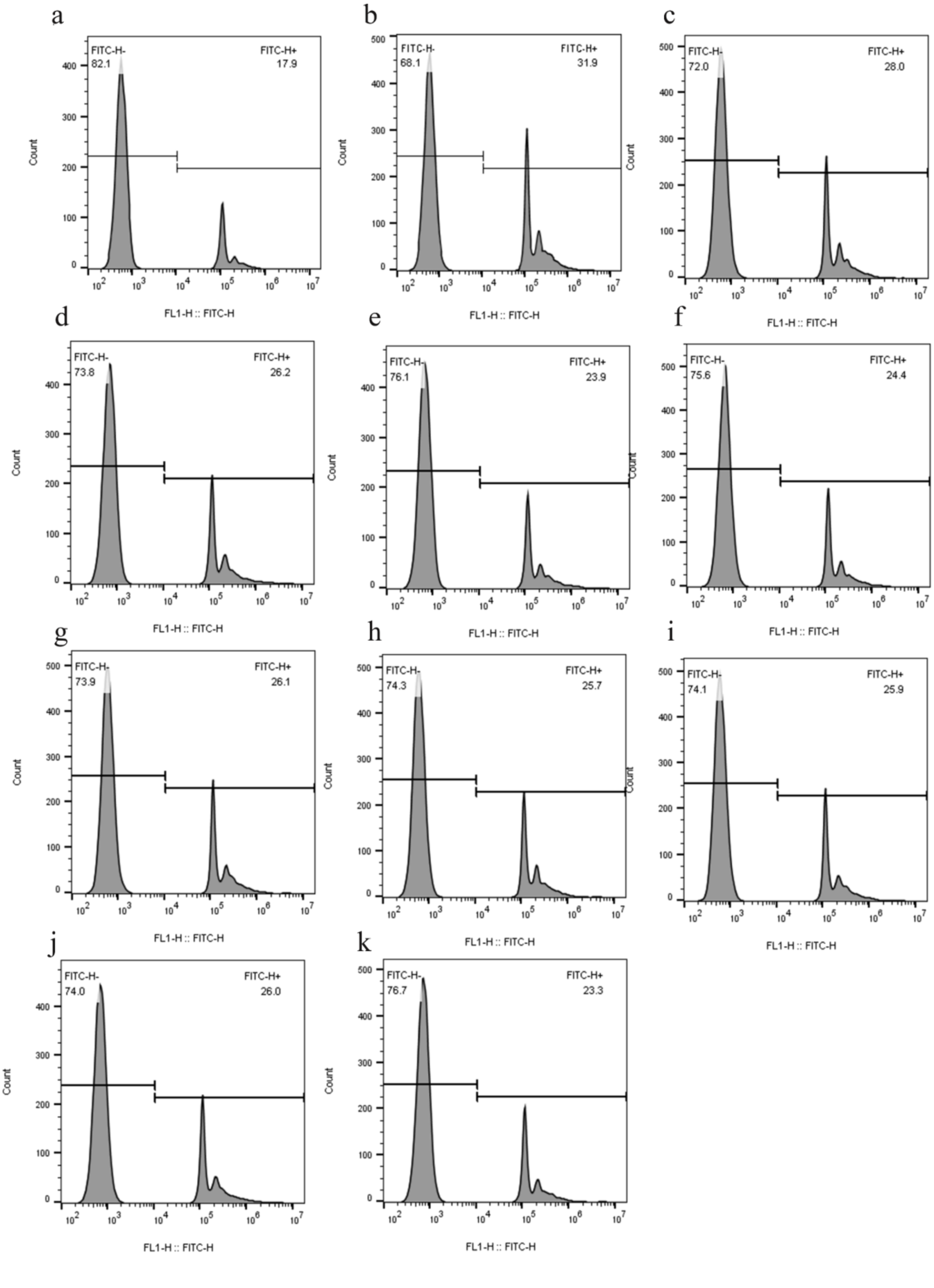

Supplementary Figure 3. Phagocytosis rates following the treatment of NACOS. a, b, c, d, e, f, g, h, i, j, and k represent the control (Ctrl), LPS, chitosan oligosaccharide (COS), low DP NACOS (LNACOS), high DP NACOS (HNACOS), NACOS1, NACOS2, NACOS3, NACOS4, NACOS5, and NACOS6, respectively.

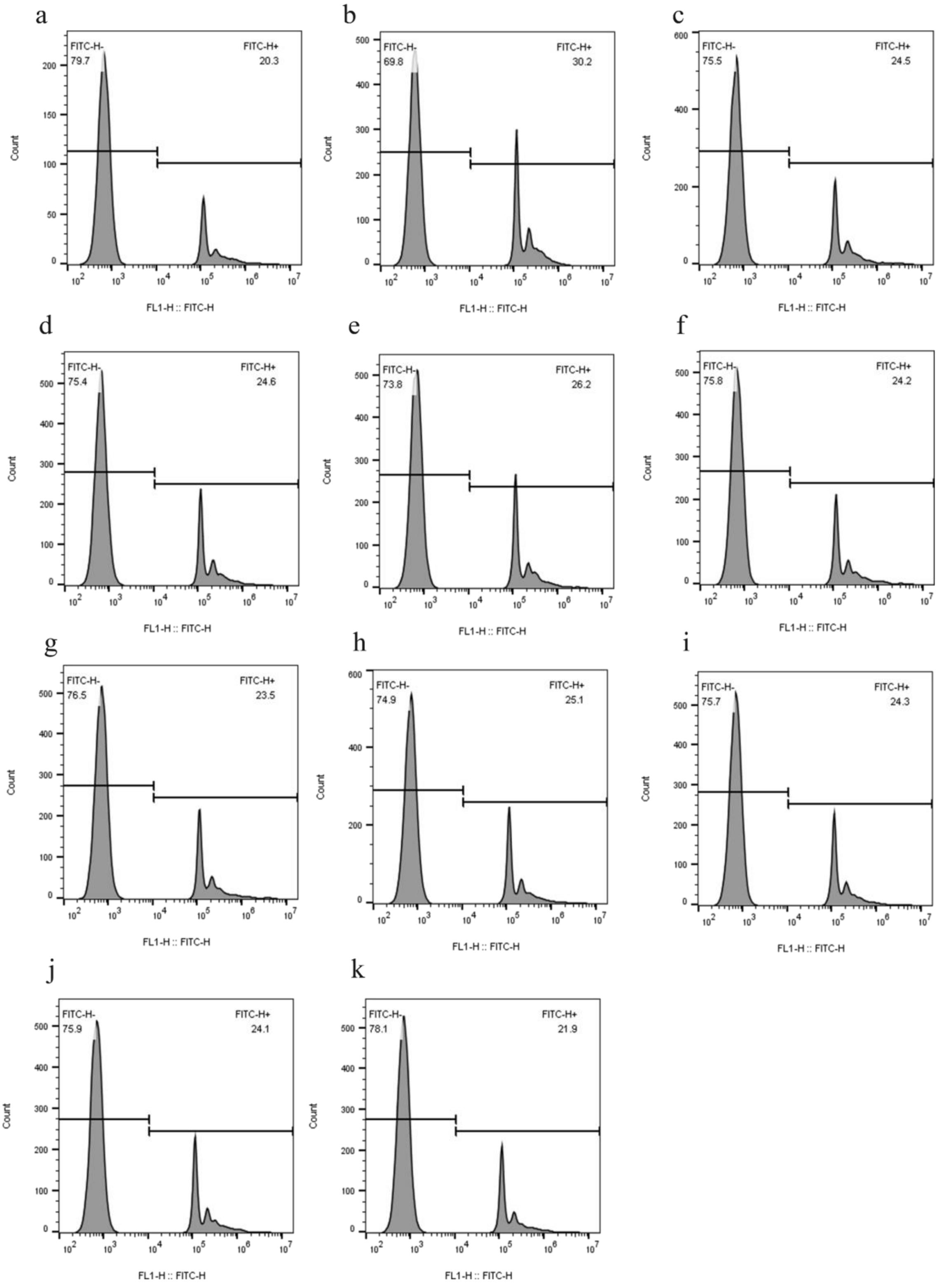

Supplementary Figure 4. The RAW264.7 phagocytosis rates in the *N*-acetyl chitooligosaccharides (NACOS) pretreated by LPS stimulation. a, b, c, d, e, f, g, h, i, j, and k represents the control (Ctrl), LPS chitosan oligosaccharide (COS), low DP NACOS (LNACOS), high DP NACOS (HNACOS), NACOS1, NACOS2, NACOS3, NACOS4, NACOS5, and NACOS6 pretreatment groups, respectively.

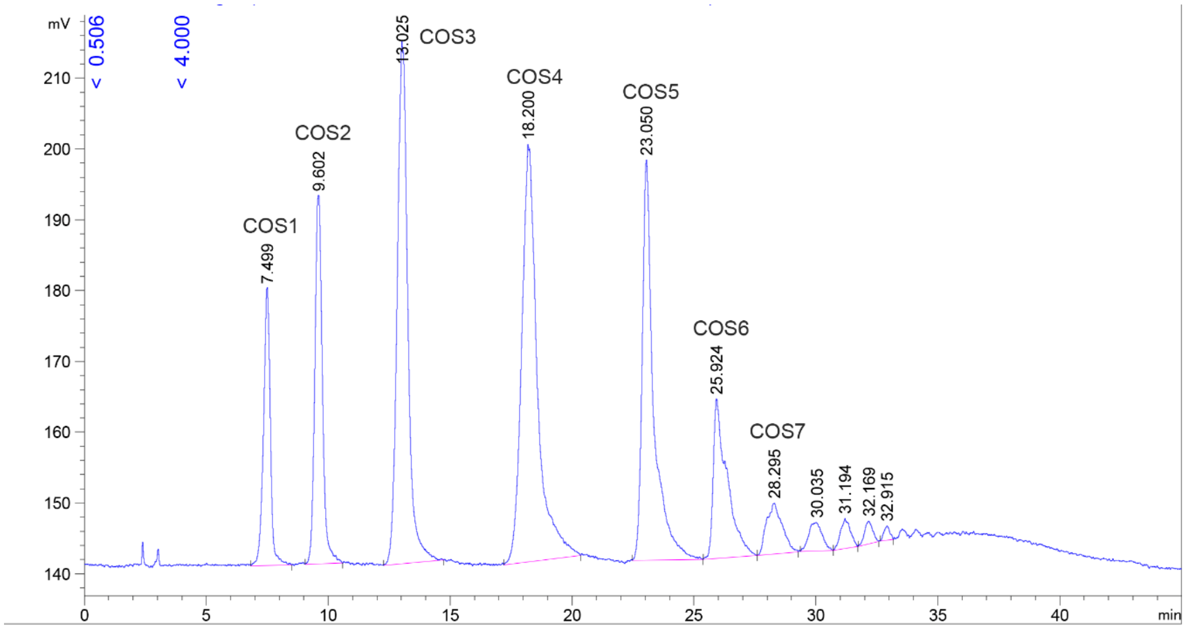

Supplementary Figure 5. High-performance liquid chromatography diagram of the chitosan oligosaccharide sample provided by Changlong Company.
